# Role of changes in SARS-CoV-2 spike protein in the interaction with the human ACE2 receptor: An *in silico* analysis

**DOI:** 10.17179/excli2020-1167

**Published:** 2020-03-18

**Authors:** Joseph Thomas Ortega, Maria Luisa Serrano, Flor Helene Pujol, Hector Rafael Rangel

**Affiliations:** 1 Department of Pharmacology and Cleveland Center for Membrane and Structural Biology, School of Medicine, Case Western Reserve University, Cleveland, OH 44106, USA; 2 Unidad de Química Medicinal, Facultad de Farmacia, Universidad Central de Venezuela, Caracas, Venezuela; 3 Laboratorio de Virología Molecular, Centro de Microbiología y Biología Celular, Instituto Venezolano de Investigaciones Científicas, Caracas, Venezuela

**Keywords:** Spike, SARS-CoV-2, ACE2, Coronavirus, outbreak

## Abstract

Many human viral diseases are a consequence of a zoonotic event. Some of the diseases caused by these zoonotic events have affected millions of people around the world, some of which have resulted in high rates of morbidity/mortality in humans. Changes in the viral proteins that function as ligands of the host receptor may promote the spillover between species. The most recent of these zoonotic events that have caused an ongoing epidemic of high magnitude is the Covid-19 epidemics caused by SARS-CoV-2. The aim of this study was to determine the mutation(s) in the sequence of the spike protein of the SARS-CoV-2 that might be favoring human to human transmission. An *in silico* approach was performed, and changes were detected in the S1 subunit of the receptor-binding domain of spike. The observed changes have significant effect on SARS-CoV-2 spike/ACE2 interaction and produce a reduction in the binding energy, compared to the one of the Bat-CoV to this receptor. The data presented in this study suggest a higher affinity of the SARS-Cov-2 spike protein to the human ACE2 receptor, compared to the one of Bat-CoV spike and ACE2. This could be the cause of the rapid viral spread of SARS-CoV-2 in humans.

## Introduction

Severe Acute Respiratory Syndrome Coronavirus (SARS-CoV), Middle Eastern Respiratory Syndrome Coronavirus (MERS-CoV), and the recently identified novel Coronavirus (SARS-CoV-2) belong to the Coronaviridae family, genus Betacoronavirus, that has been related to important epidemiological outbreaks. These are enveloped viruses with a positive-sense single-strand RNA of around 32 Kb. The viral particles contain four main structural proteins: the spike, membrane, envelope protein, and nucleocapsid. The spike protein protrudes from the envelope of the virion and plays a pivotal role in the receptor host selectivity and cellular attachment. Strong scientific evidence showed that SARS and SARS-CoV-2 spike proteins interact with angiotensin-converting enzyme 2 (ACE2) (Chen et al 2020[2]; Wan et al., 2020[16]). Also, other cellular receptors play a secondary role in the viral attachment, as the C-type lectin CD209L, and DC-SIGN binds to SARS-CoV. However, ACE2 appears to be the key functional receptor for the SARS-CoV (Coutard et al., 2020[3]; Satija and Lal, 2007[12]) and probably for SARS-CoV-2 (Walls et al., 2020[15]) The interaction between the viral protein and its cell membrane receptor is a critical step in the replication cycle. Furthermore, the efficiency of viral infection is strongly dependent on this process. Several physicochemical factors are associated with protein-protein interactions. These factors are determined by the nature of residues and the type of chemical interactions occurring between ligand and receptor. Thus, the presence of residues that produce an energetically favored interaction (lower free energy) may drive binding kinetics and finally lead to the fusion event. Based on that, this study aimed to evaluate the energetic profile of the interaction between the SARS-CoV-2 spike protein and the human cell receptor ACE2.

## Materials and Methods

### Sequence analysis

**Figure 1:**
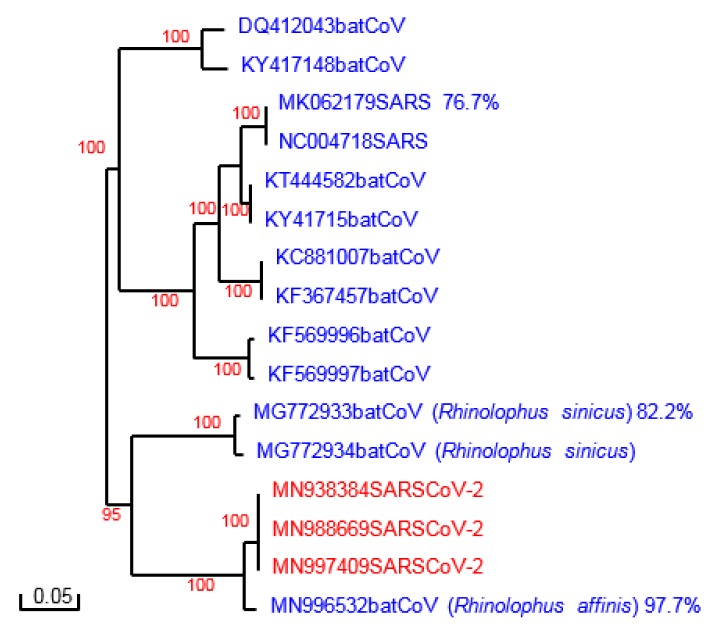
Phylogenetic analysis of SARS-CoV-2 and other coronavirus spike proteins. Phylogenetic tree constructed with Poisson correction and 100 bootstrap replicas. The sequences are named with their accession number. Percent homology with SARS-CoV-2 spike protein is shown for some proteins.

Sequences analyzed were individually retrieved from GenBank (accession numbers are shown in the phylogenetic tree, see Figure 1(Fig. 1)). The sequence used for the modeling process and construct the spike protein model of SARS-CoV2 were obtained from the protein database and correspond to 6ACC (SARS spike protein) and 6ACD (Bat-spike protein).

### Protein modeling

Homology structural models of viral spike protein from SARS-CoV-2 (QHO62112.1) and Bat-CoV (AAZ67052.1) were built by using the tools of the SWISS-MODEL modeling server and the DeepView/Swiss-PdbViewer 4.01 software (Arnold et al., 2006[1]). Several models were obtained and the quality of each structure was evaluated. The best model for SARS-CoV-2 spike was obtained using the crystal structure of SARS-CoV spike protein (PDB code 6ACC). On the other hand, for Bat-CoV, the best model was obtained using as template the crystal structure PDB code 6ACD. These models were subjected to further protein structure optimization. Hydrogen atoms were added and the partial charges were assigned for energy refinement. The protein model was embedded in a 100 Å water box. Then, energy minimization was performed while applying constraints to the protein backbone to preserve global folding and optimizing the relative position of the water molecules and protein. The obtained systems underwent MD simulations using NAMD as described by Ortega et al. (2019[8]). All MD simulations described in this study were performed with NAMD 2.12 (Phillips et al., 2005[10]), Vega ZZ 3.1.0.21 (Pedretti et al., 2004[9]). CHARMM force field (Vanommeslaeghe et al., 2010[14]) and Gasteiger charges were used. The obtained structures represent the lowest energy frame of the MD simulations. The quality of the models was established with ProSA (Wiederstein and Sippl, 2007[17]) and PROCHECK programs (Laskowski et al., 1993[4]).

### Protein-protein docking

Crystal structure for SARS-CoV spike (PDB code 6ACK) and ACE2 (PDB code 1R42) were downloaded from the Protein Data Bank. Also, the homology model for SARS-CoV-2 spike was assayed. Protein preparation was carried out as described above. Then, binding patterns and affinity estimations for the interaction between the viral spike and ACE2 receptor were performed using molecular docking. This process was performed through two steps; first, a blind docking between ligand (spike protein) and receptor (ACE2) was performed using Z-dock software (Pierce et al., 2014[11]). Then, the resulting docking data were processed and analyzed by using the tools of PRODIGY software (Xue et al., 2016[19]). Finally, results were clustered and analyzed considering binding energies and main interacting residues in each complex.

## Results

### Homology analysis of the spike proteins of SARS-CoVs and related Bat-CoVs

**Figure 2:**
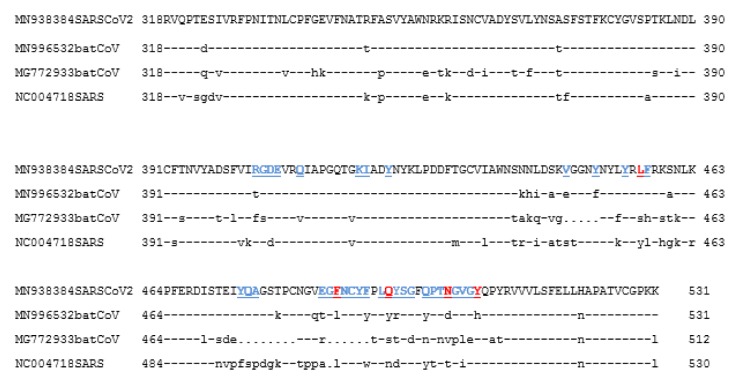
Receptor Binding Domain of the spike protein sequence alignment of SARS-CoV-2 and other related Coronaviruses. Sequence aligment for the interacting domain of SARS-CoV-2 (MN938384), Bat-CoV (MN996532 and MG772933) and SARS-CoV (NC004718). The key amino acids described for the interaction with ACE2 are shown in red, and in blue others amino acid related with the interaction in SARS-CoV2. (Lines (-) = same amino acid, dots (.) =deletion)

Phylogenetic analysis of the spike protein sequences of SARS-CoV-2 and Bat-CoVs, SARS-CoV is shown in Figure 1(Fig. 1). The results are in agreement with recent reports of an independent introduction of SARS-CoV-2 from a Bat-CoV, different from the spillover which led to the introduction of SARS-CoV, being the Bat-CoV of *Rhinolophus affinis* the probable ancestor of this new virus (Wong et al., 2020[18]). Indeed, the sequences of the whole spike of this Bat-CoV and of SARS-CoV-2 share 97.7 % identity (Figure 1(Fig. 1)). More divergence is found however in the S1 subunit, particularly in the Receptor Binding Domain (RBD) of the different spike proteins. SARS-CoV and Bat-CoV from *Rhinolophus sinicus* (originally signaled as the most closely related virus to SARS-CoV-2) exhibit several amino acid substitutions and deletions in the RBD compared to SARS-CoV-2. The RBD of Bat-CoV from *Rhinolophus affinis*, although more closely related to the one of SAS-CoV-2, also displayed several amino acid substitutions (Figure 2(Fig. 2)).

### Structural analysis of Spike-ACE2 complexes

**Figure 3:**
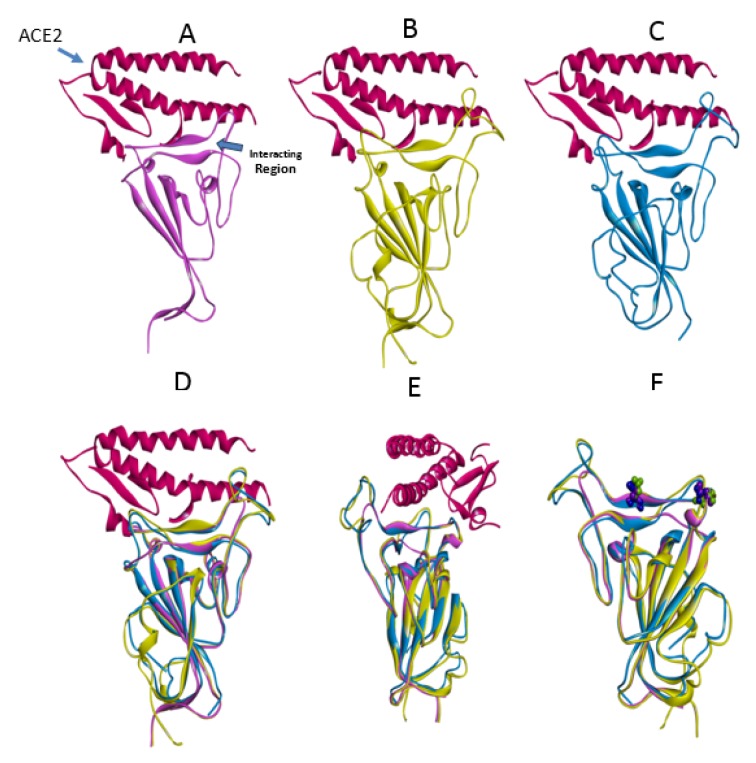
Coronavirus spike proteins. The spike proteins in complex with the RBD of ACE2 (dark pink) are shown A) Bat-CoV, B) SARS-CoV, and C) SARS-CoV-2. A comparison between the three spike proteins are shown in D and a 45 degree turn is also shown in E. The location of the main residues mutated in SARS-CoV (position 479 and 487) and SARS-CoV-2 are shown in F (green and blue).

The crystal structures of the spike protein of SARS-CoV and homology models of Bat-CoV (accession number MG772933), Bat-CoV of *Rhinolophus sinicus*, and SARS-CoV-2 interacting with the putative binding domain site in human ACE2 were analyzed. The interaction pattern between the three viral spikes is quite similar. The main region of interaction with the putative cellular receptor counter-part is formed by fifteen residues ordered into a beta-sheet conformation surrounded by two capping loops (Figure 3(Fig. 3) and Supplementary Figure 1). Interestingly, sequence comparison between SARS-CoV-2 and SARS-CoV revealed that the residues present in the receptor-interacting motive are highly conserved with 70 % identity, sharing nine residues between both viruses. In the SARS-CoV RBD are present residues that allowed the interspecies infection, known as Y442, L472, N479, D480, and T487 (Lu et al., 2015[6]). However, in SARS-CoV-2, slight modification of some residues could improve the interaction with the human cellular receptor: L455, F486, Q493, and N501. In SARS-CoV, two main residues (479 and 487) have been associated to the recognition of the human ACE2 receptor (Lu et al., 2015[6]). These residues suffered a punctual mutation from civet to human, K479N and S487T (Li, 2013[5]). In the SARS-CoV-2, the residues corresponding to N479 correspond to Q493 and T487 to N501. These changes in the SARS-CoV-2 represent energetically favorable changes for the interaction with the receptor. The local environment present in the ACE2 receptor allows these mutations to produce a significant number of electrostatic stabilizing interactions (Table 1(Tab. 1)). Furthermore, as mentioned previously, the presence of the two capping loops in the binding domain is likely producing a stabilization effect over the interaction with the cellular receptor. Our models showed that these capping loops appear in both human-infecting viruses but are absent in the bat virus. The data showed here strongly suggest that these capping loops produce an increase in the electrostatic interactions between the spike protein and the cellular receptor. In SARS-CoV, the residues present in these capping loops showing direct interaction with the receptor are R426, S432, T433, Y436, P462, D463, S472, and N473 and in SARS-CoV-2 are V445, Y449, Y473, Q474, A475, E484, G485, F486, and N487. The counter-pairs located in the ACE2 receptor are shown in Table 1(Tab. 1). Altogether, the higher number of protein-protein contacts (Table 2(Tab. 2)) and the longer capping loops could explain the increase in binding affinities in SARS-CoV-2 (-15.7 Kcal/mol) in comparison with SARS-CoV (-14.1 Kcal/mol) (Table 3(Tab. 3)). Thus, these loops could play an important role together with the punctual mutations being an interesting clue to determine the host receptor specificity for the viral spike protein.

## Discussion

Several amino acid substitutions in RBD were identified in the SARS-CoV-2 RBD compared to Bat-SARS-CoVs and SARS-CoV. Mutations in the spike protein could change the tropism of a virus, including new hosts or increasing viral pathogenesis (Shang et al., 2020[13]). HIV infection is a good model of change in viral tropism by mutations in the envelope proteins. These mutations switch co-receptor use (CCR5 to CXCR4) increasing the viral pathogenesis (Mosier 2009[7]). Interestingly, our data showed that these changes are not only related to the ability of interaction with the human ACE2 receptor but also for improving this receptor recognition. The presence of two loops around the RBD of SARS-CoV-2 might be promoting the interaction with the ACE2 receptor, improving the binding to this receptor by increasing the number of atoms involved (Tables 1(Tab. 1) and 2(Tab. 2)). The amino acid substitutions and the longer capping loops could explain the increase in binding affinities in SARS-CoV-2 compared to SARS-CoV (Table 1(Tab. 1)). Higher affinity values might be related to the dynamic of infection and the rapid spread observed for this virus.

The origin of the SARS-CoV-2 has been not fully elucidated. While this study was in course, another study of Wong et al. (2020[18]), showed a high similarity at protein level in the RBD among the coronaviruses isolated from the recent outbreak (SARS-CoV-2), those isolated from pangolin and *Rhinolophus affinis *(RaTG13)*. *The authors also suggest that Pangolin might be the intermediate host, with a 98 % identity with the human virus, at the receptor binding motif, between the bat and human. The spike model of RaTG13 is quite similar to that obtained from SARS-CoV and SARS-CoV-2 and the loops in the RBD are also present (data not shown). The protein sequence of the receptor binding motif, has 5 important amino acids. When comparing the sequence of SARS-CoV-2 with that of the isolated viruses of pangolin and *Rhinolophus affinis*, 1 and 4 differences are observed respectively in the amino acids considered key for the union with ACE2 (Yan et al., 2020[20]; Wong et al., 2020[18]). These differences should mean slightly less favorable binding energies between these viruses with ACE2 compared to the SARS-CoV-2, shown in this study. Thus, the loops observed in the spike protein of SARS-CoV-2 could play an important role together with the amino acid substitutions, being an interesting clue to determine the host receptor specificity for the viral spike protein. Altogether, structural changes and residues composition in the viral spike protein could be associated with increased infection kinetics and viral spreading. Comparative studies to determine the impact *in vitro* of the mutation and loops in RBD of SARS-CoV and SARS-CoV-2 are required in order to predict possible zoonotic event in the future.

## Supplementary Material

Supplementary information

## Figures and Tables

**Table 1:**
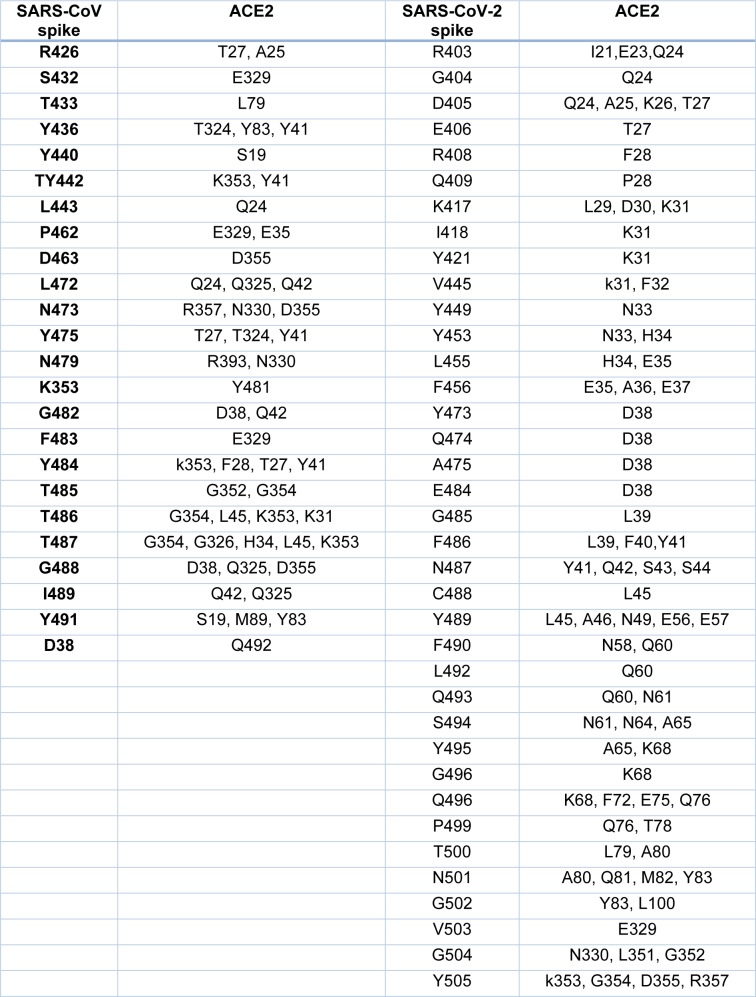
Residues involved in the Interaction between viral spike and ACE2 (SARS)

**Table 2:**
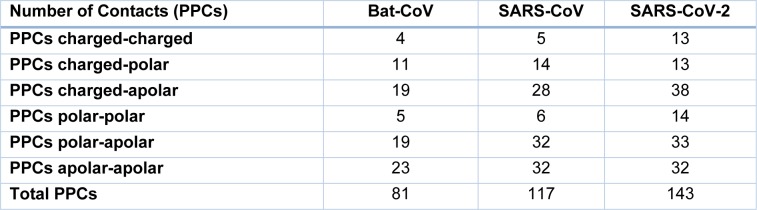
Number of protein-protein contacts (PPC) between CoV spikes and ACE2

**Table 3:**
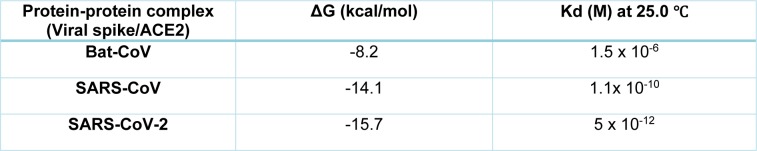
Binding affinity (ΔG) and dissociation constant (Kd) predicted values for the interaction between viral spike and ACE2 receptor

## References

[1] Arnold K, Bordoli L, Kopp J, Schwede T (2006). The SWISS-MODEL workspace: A web-based environment for protein structure homology modelling. Bioinformatics.

[2] Chen Y, Guo Y, Pan Y, Zhao ZJ (2020). Structure analysis of the receptor binding of 2019-nCoV. Biochem Biophys Res Commun.

[3] Coutard B, Valle C, de Lamballerie X, Canard B, Seidah NG, Decroly E (2020). The spike glycoprotein of the new coronavirus 2019-nCoV contains a furin-like cleavage site absent in CoV of the same clade. Antiviral Res.

[4] Laskowski RA, MacArthur MW, Moss DS, Thornton JM (1993). PROCHECK - a program to check the stereochemical quality of protein structures. J Appl Crystallogr.

[5] Li F (2013). Receptor recognition and cross-species infections of SARS coronavirus. Antiviral Res.

[6] Lu G, Wang Q, Gao GF (2015). Bat-to-human: spike features determining 'host jump' of coronaviruses SARS-CoV, MERS-CoV, and beyond. Trends Microbiol.

[7] Mosier DE (2009). How HIV changes its tropism: evolution and adaptation?. Curr Opin HIV AIDS.

[8] Ortega JT, Serrano ML, Suárez AI, Baptista J, Pujol FH, Cavallaro LV (2019). Antiviral activity of flavonoids present in aerial parts of Marcetia taxifolia against Hepatitis B virus, Poliovirus, and Herpes simplex virus in vitro. EXCLI J.

[9] Pedretti A, Villa L, Vistoli G (2004). VEGA - An open platform to develop chemo-bio-informatics applications, using plug-in architecture and script programming. J Comput Aided Mol Des.

[10] Phillips JC, Braun R, Wang W, Gumbart J, Tajkhorshid E, Villa E (2005). Scalable molecular dynamics with NAMD. J Comput Chem.

[11] Pierce BG, Wiehe K, Hwang H, Kim B-H, Vreven T, Weng Z (2014). ZDOCK server: interactive docking prediction of protein–protein complexes and symmetric multimers. Bioinformatics.

[12] Satija N, Lal SK (2007). The molecular biology of SARS coronavirus. Ann NY Acad Sci.

[13] Shang J, Wan Y, Liu C, Yount B, Gully K, Yang Y (2020). Structure of mouse coronavirus spike protein complexed with receptor reveals mechanism for viral entry. PLoS Pathog.

[14] Vanommeslaeghe K, Hatcher E, Acharya C, Kundu S, Zhong S, Shim J (2010). CHARMM general force field: A force field for drug-like molecules compatible with the CHARMM all-atom additive biological force fields. J Comput Chem.

[15] Walls AC, Park YJ, Tortorici MA, Wall A, McGuire AT, Veesler D (2020). Structure, function, and antigenicity of the SARS-CoV-2 spike glycoprotein. Cell.

[16] Wan Y, Shang J, Graham R, Baric RS, Li L (2020). Receptor recognition by novel coronavirus from Wuhan: An analysis based on decade-long structural studies of SARS. J Virol.

[17] Wiederstein M, Sippl MJ (2007). ProSA-web: interactive web service for the recognition of errors in three-dimensional structures of proteins. Nucleic Acids Res.

[18] Wong MC, Javornik Creegen SJ, Ajami NJ, Petrosino JF (2020). Evidence of recombination in coronaviruses implicating pangolin origins of nCoV-2019. bioRxiv.

[19] Xue LC, Rodrigues JP, Kastritis PL, Bonvin AM, Vangone A (2016). PRODIGY: a web server for predicting the binding affinity of protein-protein complexes. Bioinformatics.

[20] Yan R, Zhang Y, Li Y, Xia L, Guo Y, Zhou Q (2020). Structural basis for the recognition of the SARS-CoV-2 by full-length human ACE2. Science.

