# Supplementary material for: Role of changes in SARS-CoV-2 spike protein in the interaction with the human ACE2 receptor: An in silico analysis
Source: EXCLI J. 2020 Mar 18;19:410–7. doi: 10.17179/excli2020-1167 (PMC7081066; doi:10.17179/excli2020-1167)
Supplement: Supplementary information [file EXCLI-19-410-s-001.pdf]

EXCLI Journal 2020;19:410-417 – ISSN 1611-2156
Received: February 25, 2020, accepted: March 16, 2020, published: March 18, 2020

Supplementary information to:

ROLE OF CHANGES IN SARS-COV-2 SPIKE PROTEIN IN THE INTERACTION WITH THE HUMAN ACE2 RECEPTOR: AN *IN SILICO* ANALYSIS

Joseph Thomas Ortega[1], Maria Luisa Serrano[2], Flor Helene Pujol[3], Hector Rafael Rangel[3]*

1 Department of Pharmacology and Cleveland Center for Membrane and Structural Biology, School of Medicine, Case Western Reserve University, Cleveland, OH 44106, USA
2 Unidad de Química Medicinal, Facultad de Farmacia, Universidad Central de Venezuela, Caracas, Venezuela
3 Laboratorio de Virología Molecular, Centro de Microbiología y Biología Celular, Instituto Venezolano de Investigaciones Científicas, Caracas, Venezuela

* **Corresponding author:** Hector Rafael Rangel, Laboratorio de Virología Molecular, Centro de Microbiología y Biología Celular, Instituto Venezolano de Investigaciones Científicas, Caracas, Venezuela; E-mail: hrangel2006@gmail.com

http://dx.doi.org/10.17179/excli2020-1167

This is an Open Access article distributed under the terms of the Creative Commons Attribution License (http://creativecommons.org/licenses/by/4.0/).

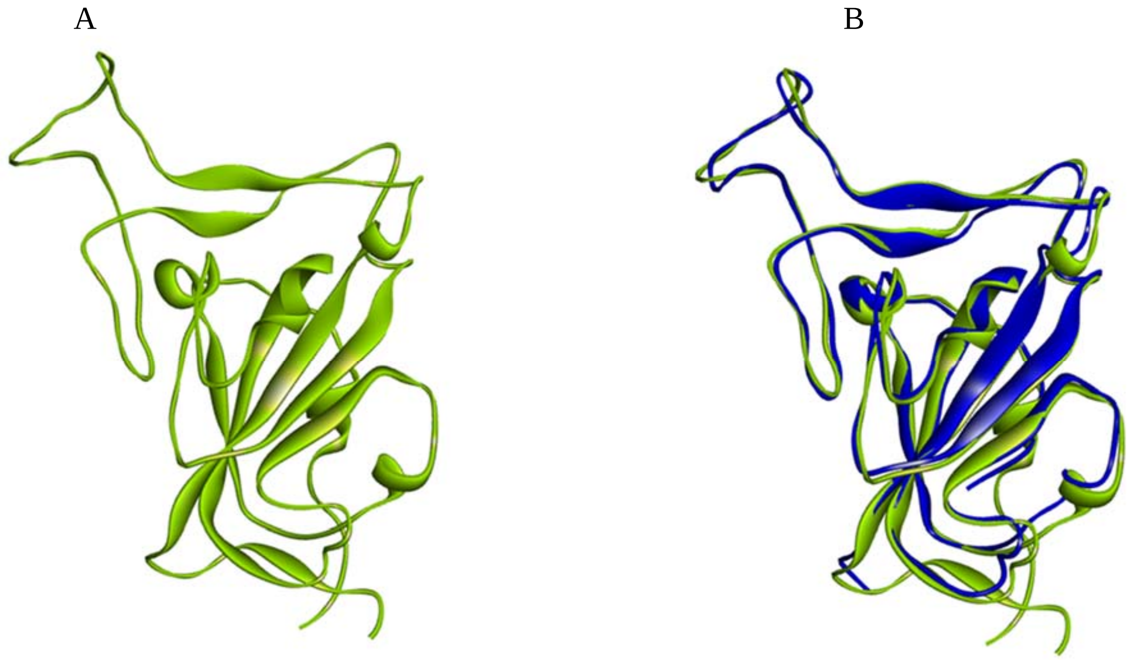

Supplementary Figure 1: Spike protein of *Rhinolophus sinicus* (**A**) *and the merge between spike of Rhinolophus sinicus and* SARS-CoV (**B**). The merge image shows a high level of structural similarity in both protein, suggesting a similar pattern of interaction with the receptor.
